# Association between oxidative stress, mitochondrial function of peripheral blood mononuclear cells and gastrointestinal cancers

**DOI:** 10.1186/s12967-023-03952-8

**Published:** 2023-02-10

**Authors:** Weili Liu, Yuan Gao, Hua Li, Xinxing Wang, Min Jin, Zhiqiang Shen, Dong Yang, Xuelian Zhang, Zilin Wei, Zhaoli Chen, Junwen Li

**Affiliations:** 1 Tianjin Institute of Environmental and Operational Medicine, Tianjin, China; 2 Maternity & Child Care Center of Dezhou, Dezhou, Shandong China; 3 Department of Endoscopy, National Clinical Research Center for Cancer, Key Laboratory of Cancer Prevention and Therapy, Tianjin’s Clinical Research Center for Cancer, Tianjin Medical University Cancer Institute and Hospital, Tianjin, China

**Keywords:** Gastrointestinal cancers, PBMCs, Mitochondrial dysfunction

## Abstract

**Background:**

The incidence and mortality rate of gastrointestinal cancers are high worldwide. Increasing studies have illustrated that the occurrence, progression, metastasis and prognosis of cancers are intimately linked to the immune system. Mitochondria, as the main source of cellular energy, play an important role in maintaining the physiological function of immune cells. However, the relationship between mitochondrial function of immune cells and tumorigenesis has not yet been systematically investigated.

**Methods:**

A total of 150 cases, including 60 healthy donors and 90 primary gastrointestinal cancer patients without anti-tumor treatments (30 with gastric cancer, 30 with liver cancer and 30 with colorectal cancer) were involved in our study. The oxidant/antioxidant and cytokine levels in plasma, the ROS level, mitochondrial function and apoptosis ratio of peripheral blood mononuclear cells (PBMCs) were evaluated.

**Results:**

The imbalance between oxidant and antioxidant in plasma was discovered in the primary gastrointestinal cancer patients. The levels of cell reactive oxygen species (ROS) and mitochondrial ROS in PBMCs of primary gastrointestinal cancers were significantly increased compared with that in healthy donors. Meanwhile, the ATP content, the mtDNA copy number and the mitochondrial membrane potential (MMP) in PBMCs of patients with primary gastrointestinal cancers were lower than those in control group. The decreased MMP also occurred in immune cells of gastrointestinal cancers, including T cell, B cell, NK cell and monocyte. Furthermore, the PBMCs apoptosis ratio of primary gastrointestinal cancer patients was significantly higher than that of control group. Importantly, an increase of IL-2 and IL-6 and a decrease of IgG in plasma were found in the patients with primary gastrointestinal cancers. These changes of mitochondrial function in immune cells were consistent among primary gastrointestinal cancers without anti-tumor treatments, such as liver cancer, gastric cancer and colorectal cancer.

**Conclusion:**

Our study demonstrated that the imbalance of oxidation/antioxidation in primary gastrointestinal cancer patients without anti-tumor treatments results in excessive ROS. The oxidative stress was associated to the mitochondrial dysfunction, the apoptosis of immune cells and eventually the abnormal immune function in primary gastrointestinal cancers. The application of immune cell mitochondrial dysfunction into clinical evaluation is anticipated.

## Background

In 2021, Sung et al. published the global cancer statistics of 2020 and provided the estimates of incidence and mortality worldwide for 36 cancers in 185 countries [1]. This research reported that the number of new cancer cases was estimated to be 19,292,789 in the world with a gross incidence rate of 247.5/10 million in 2020. The number of new deaths was 9,958,133 cases and the crude mortality rate was 127.8/10 million. Top five types of cancer with incidence were breast cancer (11.7%), lung cancer (11.4%), colorectal cancer (10%), prostate cancer (7.3%) and gastric cancer (5.6%). Lung cancer is the leading cause of cancer death worldwide, accounting for 18% of the total cancer deaths, followed by colorectal cancer (9.4%), liver cancer (8.3%), gastric cancer (7.7%) and female breast cancer (6.9%). Therefore, the impact of gastrointestinal cancers on human health cannot be ignored.

With advances of tumor immunology and molecular biology, researchers have realized that the occurrence and development of malignant tumors are not only related to the change of the genetic background of tumor cells, but also closely related to the immune function of the body. Immunologists put forward the theory of tumor immune editing, including immune surveillance and immune escape [2]. In recent years, it has been demonstrated that the occurrence, development, metastasis and prognosis of tumor are significantly associated with the immune mechanism of the body. The decline of immune function can lead to the occurrence of tumor [3, 4]. For instance, patients with lung cancer had varying degrees of reduction of T lymphocyte subsets in peripheral blood, especially in patients with advanced cancer [5]. Analogously, a study about liver cancer found that the cellular immune function of patients was generally lower than that of healthy persons. CD3^+^ T cells, CD4^+^ T cells and CD4^+^/ CD8^+^ T were decreased in the liver cancer patients. These results suggested that the obvious changes of cellular immunosuppression occurred in the occurrence and development of liver cancer [6, 7].

Recently, the relationship between cancer and mitochondria functions has gained more attention. It has been revealed that the mitochondrial electron transport chain is necessary for tumor growth [8, 9]. As is well known, the basic function of mitochondria is to synthesize ATP and supply energy for cells [10]. Mitochondria ensure the needs of almost all cellular physiological processes by producing ATP, such as the contraction of skeletal muscle and myocardium, the maintenance of transmembrane ion gradient, and even the secretion of hormones and neurotransmitters [11]. A large number of studies have shown that mitochondrial abnormalities are closely related to various diseases such as neurodegenerative diseases, diabetes and cardiovascular diseases [12]. However, the role of mitochondrial changes of immune cells in tumor development has not been fully elucidated. The biological function of immune cells directly or indirectly depends on the energy supply of ATP [13, 14]. As the main source of cell energy, mitochondria play an important role in maintaining the normal physiological function of cells. Also, mitochondria is involved in the production of oxygen free radicals and regulation of cell death process.

Therefore, we hypothesized that mitochondrial dysfunction of peripheral blood mononuclear cells (PBMCs) was associated with the gastrointestinal cancers. The present study aimed to verify the hypothesis by investigating whether PBMCs mitochondrial function in patients with gastrointestinal cancers is lower than that in healthy controls using a case-control study.

## Methods

### Participants

A total of 90 patients with gastrointestinal cancers, including 30 with gastric cancer, 30 with liver cancer and 30 with colorectal cancer, were enrolled in the study (Table 1). The inclusion criteria for cancer patients are as follows: (1) Patients with complete clinical data voluntarily participated in the study; (2) no history of chronic diseases of blood or immune system; (3) no previous history of malignant tumors; (4)These patients were first diagnosed as primary gastric cancer, liver cancer or colorectal cancer, excluding metastatic gastrointestinal cancers; (5) The gastrointestinal cancers were confirmed by pathology or histology; (6) The peripheral blood samples were collected for subsequent evaluation before anti-tumor treatments, such as radiotherapy, chemotherapy, surgical treatment, immunotherapy, etc.; and (7) the confounding factors such as gender, age, long-term residence, smoking, drinking and lifestyle of cancer patients were matched with those of healthy controls. Meanwhile, 60 healthy donors were recruited as the control group. Inclusion of healthy volunteers requires: (1) healthy volunteers voluntarily participated in the study; (2) no history of chronic diseases of blood or immune system; (3) no history of malignant tumors; (4) the gender, age, long-term residence, smoking, drinking, lifestyle and other confounding factors of healthy volunteers were matched with the case group.

**Table 1:** Information of gastrointestinal cancer patients and healthy donors

	Gastrointestinal cancers	Control
Numbers	90	60
Age (years)		
Average age	56.68	57.20
Age range	43–77	40–75
Gender (%)		
Male	54 (60)	36 (60)
Female	36 (40)	24 (40)
Tumor type (%)		
Gastric cancer	30 (33.33)	
Liver cancer	30 (33.33)	
Colorectal cancer	30 (33.33)	

### Detection of oxidative stress parameter

The peripheral blood samples were collected before anti-tumor treatments, such as radiotherapy, chemotherapy, surgical treatment, immunotherapy, etc. The blood was anticoagulated with EDTA, followed by centrifugation for 10 min at 750×*g*. The supernatant was collected as plasma. The content of malondialdehyde (MDA), the total antioxidant capacity (T-AOC) and the glutathione peroxidase (GSH-Px) activity in plasma were detected by the assay kit (CAT#: A003-2, A015-2 and A006-2, Nanjing Jiancheng Bioengineering Institute) according to the manufacturer’s instructions.

### Cytokine level assessment

The Interleukin-2 (IL-2), Interleukin-6 (IL-6), Immunoglobulin G(IgG), tumor necrosis factor-α (TNF- α) and tumor necrosis factor-α (TNF- β) in plasma were determined by the ELISA kit (Invitrogen, USA) according to the manufacturer’s instructions.

### Preparation of peripheral blood mononuclear cells (PBMCs)

The peripheral blood samples were collected before anti-tumor treatments, such as radiotherapy, chemotherapy, surgical treatment, immunotherapy, etc. The blood was anticoagulated with EDTA, followed by centrifugation for 10 min at 750×*g*. After discarding the supernatant, an equal volume of normal saline was added and mixed gently to resuspend the peripheral blood cells. The same volume of lymphocyte separation solution was added to another centrifuge tube. And the peripheral blood suspension was also added to it gently. The tube was centrifuged at 750×*g* for 15 min, the intermediate PBMC layer was collected into another centrifuge tube, and 3 mL of normal saline was added, followed by centrifugation for 5 min at 750×*g*. After discarding the supernatant, the precipitate was used for the following detection.

### Reactive oxygen species (ROS) assessment

After the PBMCs were extracted, the cell concentration was adjusted to 1 × 10^6^/mL. The cells were resuspended in a 10 mL DCFH-DA probe (CAT#: S0033M, Beyotime) with a concentration of 10 µmol/L. Then, the cells were incubated at 37 °C for 20 min, washed twice with phosphatebuffered saline (PBS), and resuspended in 500 µL of PBS. DCFH-DAlabeled cells were analyzed using flow cytometry.

### Mitochondrial reactive oxygen species (ROS) assessment

Mitochondrial ROS (mtROS) generation was detected using MitoSOX Red (CAT#: M36008, Invitrogen). Hank’s balanced salt solution (HBSS) buffer was used to dilute the MitoSOX Red mitochondrial superoxide indicator (5 mM) to achieve a final 5 µM working solution. The PBMCs were incubated with dye for 30 min at 37 °C, washed twice with phosphatebuffered saline (PBS), and resuspended in 500 µL of PBS. MitoSOX Redlabeled cells were analyzed using flow cytometry.

### Mitochondrial membrane potential (ΔΨm) assessment

T cells and B cells in PBMCs were labelled with anti-CD3 and anti-CD19 antibodies. NK cells were labelled with anti-CD3 and anti-CD56 antibodies. Monocytes were labelled with anti-CD14 antibody. The cells were stained with 1 µM rhodamine-123 (CAT#: C2008S, Beyotime) for 30 min to evaluate the ΔΨm. The cells were then washed and analyzed using flow cytometry.

### ATP assay

Intracellular ATP of PBMCs was detected by using an ATP assay kit (CAT#: S0027, Beyotime). Briefly, cell lysis buffer was used to dissociate the PBMCs before centrifugation at 4 °C for 10 min at 12,000×*g*. The supernatant was retained for the ATP assay, in which luminance was measured by using a Synergy HT multi-mode microplate reader (Biotek, Winooski, VT, USA).

### The mtDNA copy number assessment

Genomic DNA was extracted from PBMCs using the blood/cell genomic DNA extraction kit (TIANGEN biotenology) according to the manufacturer’s protocol. The copy number of mtDNA was evaluated using quantitative real-time polymerase chain reaction (PCR). The primers used for mitochondrial encoded NADH dehydrogenase-1 (ND-1) and β-actin were as follows: ND-1 forward 5′-CCCTAAAACCCGCCACATCT-3′; ND-1 reverse 5′-GAGCGATGGTGAGAGCTAAGGT-3′; β-actin forward 5′-AAGACCCCAGCACACTTAGCC- 3′; and, HGB reverse 5′-TAGCACAGCCTGGATAGCAAC-3′.

### Flow cytometry (FCM) analysis of apoptosis

PBMCs apoptosis was measured using FCM with Propidium Iodide (PI) and Annexin V-FITC Apoptosis Detection Kits (CAT#: 556570, BD Biosciences) according to the manufacturer’s instruction. The apoptosis ratio was the sum of cells that were positive for Annexin V-FITC staining plus cells that were positive for both Annexin V-FITC and PI, divided by the total number of cells.

### Statistical analysis

SPSS 24.0 (IBM Corp., Armonk, NY, USA) was used for the statistical analysis. Data are expressed as mean ± standard deviation (SD). Two-sided *P* value of less than 0.05 was considered to indicate statistical significance.

## Results

### The imbalance of oxidation and antioxidation was observed in the primary gastrointestinal cancer patients without anti-tumor treatments

**Fig. 1:**
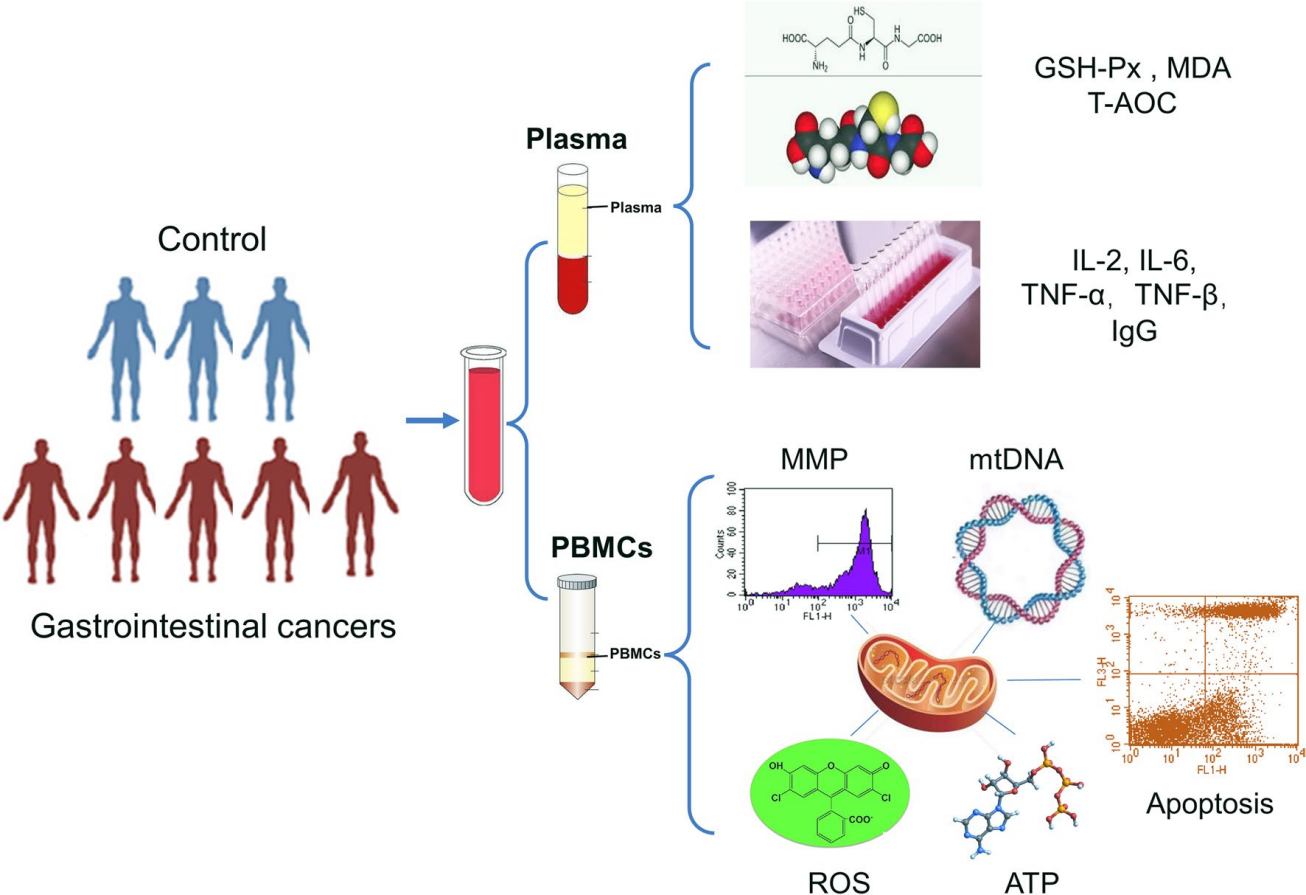
Overview of the study design. A total of 150 cases, including 60 controls and 90 primary gastrointestinal cancer patients without anti-tumor treatments (30 with gastric cancer, 30 with liver cancer and 30 with colorectal cancer) were involved in our study. Plasma was isolated to detect the oxidative stress parameter and the levels of cytokines. PBMCs was isolated to detect the level of ROS, mtROS, MMP, ATP content, the mtDNA copy number and apoptosis. PBMCs, peripheral blood mononuclear cells; T-AOC, total antioxidant capacity; GSH-Px, glutathione peroxidase; MDA, malondialdehyde; IL-2, interleukin 2; IL-6, interleukin 6; TNF-α, tumor necrosis factor α; TNF-β, tumor necrosis factor β; IgG, immunoglobulin G; MMP, mitochondrial membrane potential; ROS, reactive oxygen species; ATP, adenosine triphosphate

**Fig. 2:**
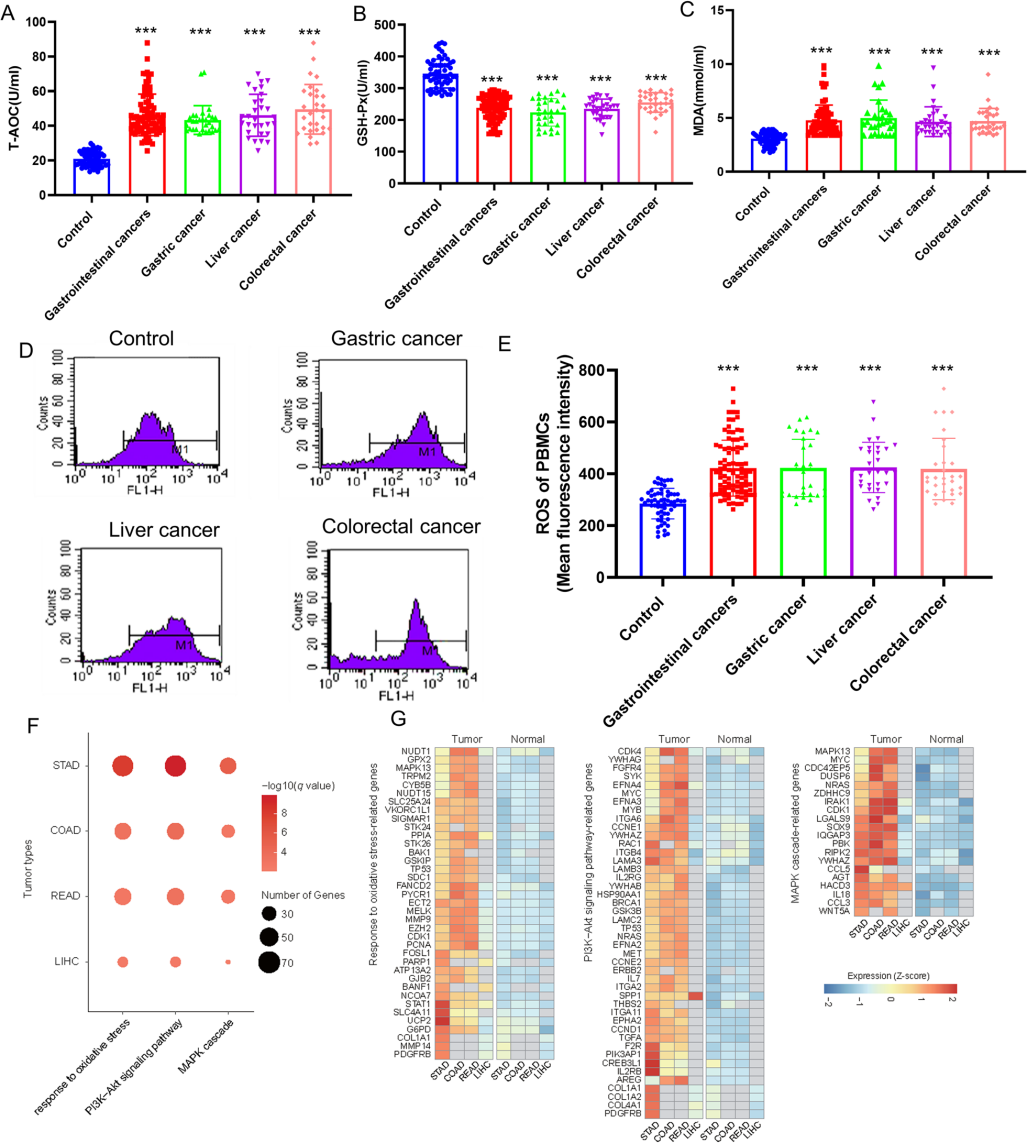
The oxidant and antioxidant level of the primary gastrointestinal cancer patients without anti-tumor treatments and healthy people. **A** The level of T-AOC of plasma in primary gastrointestinal cancer patients diagnosed for the first time and healthy people. **B** The GSH-Px activity of plasma in primary gastrointestinal cancer patients diagnosed for the first time and healthy people. **C** The content of MDA of plasma in primary gastrointestinal cancer patients diagnosed for the first time and healthy people. **D** The PBMCs ROS level were detected by flow cytometric analysis. **E** The level of PBMCs ROS in primary gastrointestinal cancer patients diagnosed for the first time and healthy people. **F** Ontology terms and KEGG pathways of the up-regulated genes between four cancer types and their paired normal tissues. **G** Heatmaps illustrating the average expression of these pathways-related genes in tumors and paired normal tissues. Values in the graphs represent the mean ± standard deviation; the symbol (***) indicates a significant increase compared with the control group (*P* < 0.001)

A total of 150 cases, including 60 healthy donors and 90 patients with primary gastrointestinal cancers without anti-tumor treatments (30 with gastric cancer, 30 with liver cancer and 30 with colorectal cancer) were included in our study (Fig. 1). Increased oxidative stress, defined as an imbalance between prooxidants and antioxidants, is associated with numerous pathophysiological processes, resulting in molecular damage and disruption of redox signaling. We examined the oxidation/antioxidation indicators in patients with primary gastrointestinal cancers (without anti-tumor treatments) and healthy controls. Compared with the controls, the total antioxidant capacity (T-AOC) and glutathione peroxidase (GSH-Px) of patients with gastrointestinal cancers were significantly decreased (Fig. 2A, B). Besides the total of primary gastrointestinal cancers, all the three gastrointestinal cancer subtypes, including gastric cancer, liver cancer and colorectal cancer, presented the consistent changes (Fig. 2A, B). While the level of malondialdehyde (MDA) of primary gastrointestinal cancers was remarkably higher than that of control groups (Fig. 2C). These results implied that the T-AOC, GSH-Px and MDA were related to primary gastrointestinal cancers without anti-tumor treatments. Notably, there were no significant differences among three caner types that are liver cancer, gastric cancer and colorectal cancer. Furthermore, we discovered that the level of reactive oxygen species (ROS) in PBMCs of primary gastrointestinal cancers without anti-tumor treatments was significantly increased compared with that of control group (Fig. 2D, E). To explore the association of ROS with oncogenic pathways, we investigated the gene expression variation in primary gastrointestinal cancers based on the TCGA datasets [15]. Four cancer types from the TCGA database were used in the differential expression analysis, including stomach adenocarcinoma (STAD), colon adenocarcinoma (COAD), rectum adenocarcinoma (READ), liver hepatocellular carcinoma (LIHC). Differentially expressed genes were identified using the LIMMA method of GEPIA2 [16] between STAD, COAD, READ and LIHC tumors and their paired normal samples, respectively. Gene ontology and KEGG pathway analysis showed that up-regulated genes in four gastrointestinal tumor tissues were significantly enriched in PI3K-Akt and MAPK signaling pathway (Fig. 2F). The average expression of these signaling-related genes were significantly increased in four cancers (Fig. 2G). These results demonstrate that increased ROS may be associated positively with the oncogenic pathways such as MAPK, PI3K, etc.

### Mitochondrial dysfunction of PBMCs was observed in the primary gastrointestinal cancer patients without anti-tumor treatments

**Fig. 3:**
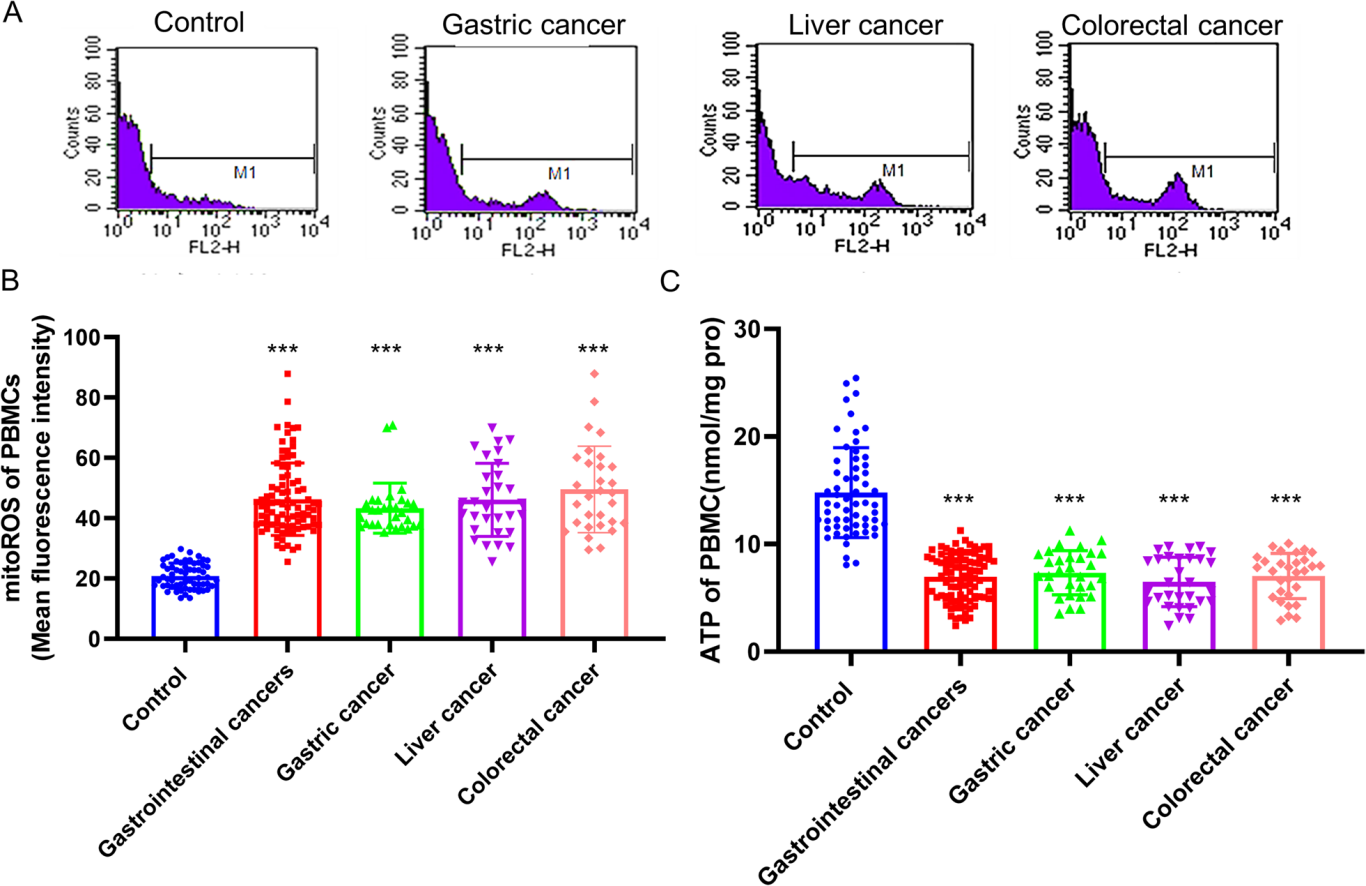
The mitoROS and ATP content of PBMCs in the primary gastrointestinal cancer patients without anti-tumor treatments and healthy people. **A** The PBMCs mitoROS level were detected by flow cytometric analysis. **B** The level of mitoROS of PBMCs in the primary gastrointestinal cancer patients diagnosed for the first time and healthy people. **C** The content of ATP of PBMCs in the primary gastrointestinal cancer patients diagnosed for the first time and healthy people. Values in the graphs represent the mean ± standard deviation; the symbol (***) indicates a significant increase compared with the control group (*P* < 0.001)

Next, we attempted to resolve whether upregulation of oncogenic signaling pathway by oxidative stress could lead to the changes in the immune repertoire. Mitochondria are at the crossroads of several cellular activities such as energy production in the form of adenosine triphosphate (ATP) through oxidative phosphorylation pathway and generation of ROS. Hence, we detected the mitochondrial function of immune cells in the primary gastrointestinal cancer patients without anti-tumor treatments and healthy donors. The mtROS level in the PBMCs of patients with primary gastrointestinal cancers was higher than that in the control group (Fig. 3A, B). Meanwhile, the ATP of PBMCs was markedly reduced in PBMCs from the patients with gastrointestinal cancers compared with that in the control group (Fig. 3C). These changes were conserved between three gastrointestinal cancer subtypes. Also, it has been reported that the mtDNA copy number, an index of mitochondrial biogenesis, is extensively changed under the pathophysiological condition. The copy number of mtDNA-encoded subunit ND-1 (mtND-1) is a convenient and well-recognized marker for total mtDNA copy number. The mtND-1 copy number in PBMCs of the primary gastrointestinal cancer patients without anti-tumor treatments was lower than that in the control group (Table 2). There were no significant differences of ATP, mtROS and mtND-1 in PBMCs between liver cancer, gastric cancer and colorectal cancer. These results suggested that mitochondrial dysfunction was observed in PBMCs of the primary gastrointestinal cancers patients without anti-tumor treatments.

**Table 2:** The mtND-1 copry number in peripheral blood mononuclear cells of the normal controls and primary gastrointestinal cancer patients without anti-tumor treatments (*x ± s*)

Group	n	mtND-1	P***
Control	60	4.84 ± 1.90	
Gastrointestinal cancers	90	1.12 ± 0.56	< 0.001
Gastric cancer	30	0.93 ± 0.64	< 0.001
Liver cancer	30	1.22 ± 0.60	< 0.001
Colorectal cancer	30	1.21 ± 0.39	< 0.001

*** *P* < 0.01 VS Control

### Mitochondrial membrane potential (ΔΨm) of multiple immune cells was significantly decreased in the primary gastrointestinal cancer patients without anti-tumor treatments

**Fig. 4:**
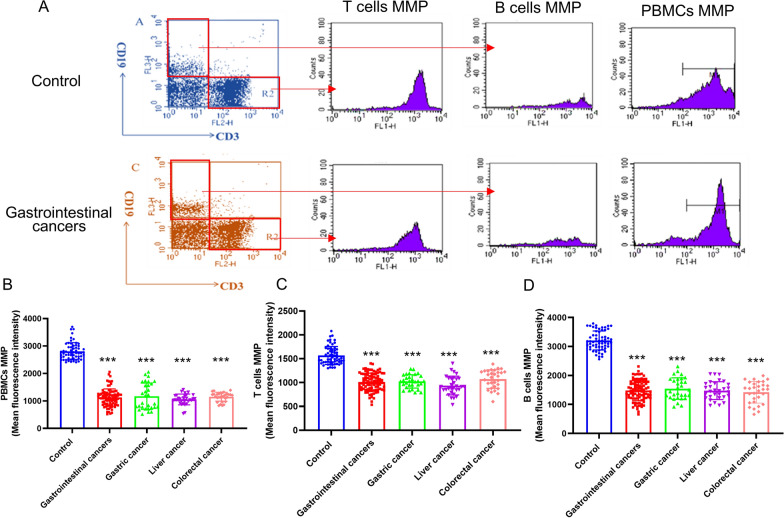
The mitochondrial membrane potential (MMP) of T cells and B cells in the primary gastrointestinal cancer patients without anti-tumor treatments and healthy people. **A** The MMP level were detected by flow cytometric analysis. **B** The MMP level of T cells (CD3^+^CD19^−^) in the primary gastrointestinal cancer patients diagnosed for the first time and healthy people. **C** The MMP level of B cells (CD3^−^CD19^+^) in the primary gastrointestinal cancer patients diagnosed for the first time and healthy people. **D** The MMP level of PBMCs in the primary gastrointestinal cancer patients diagnosed for the first time and healthy people. Values in the graphs represent the mean ± standard deviation; the symbol (***) indicates a significant increase compared with the control group (*P* < 0.001)

**Fig. 5:**
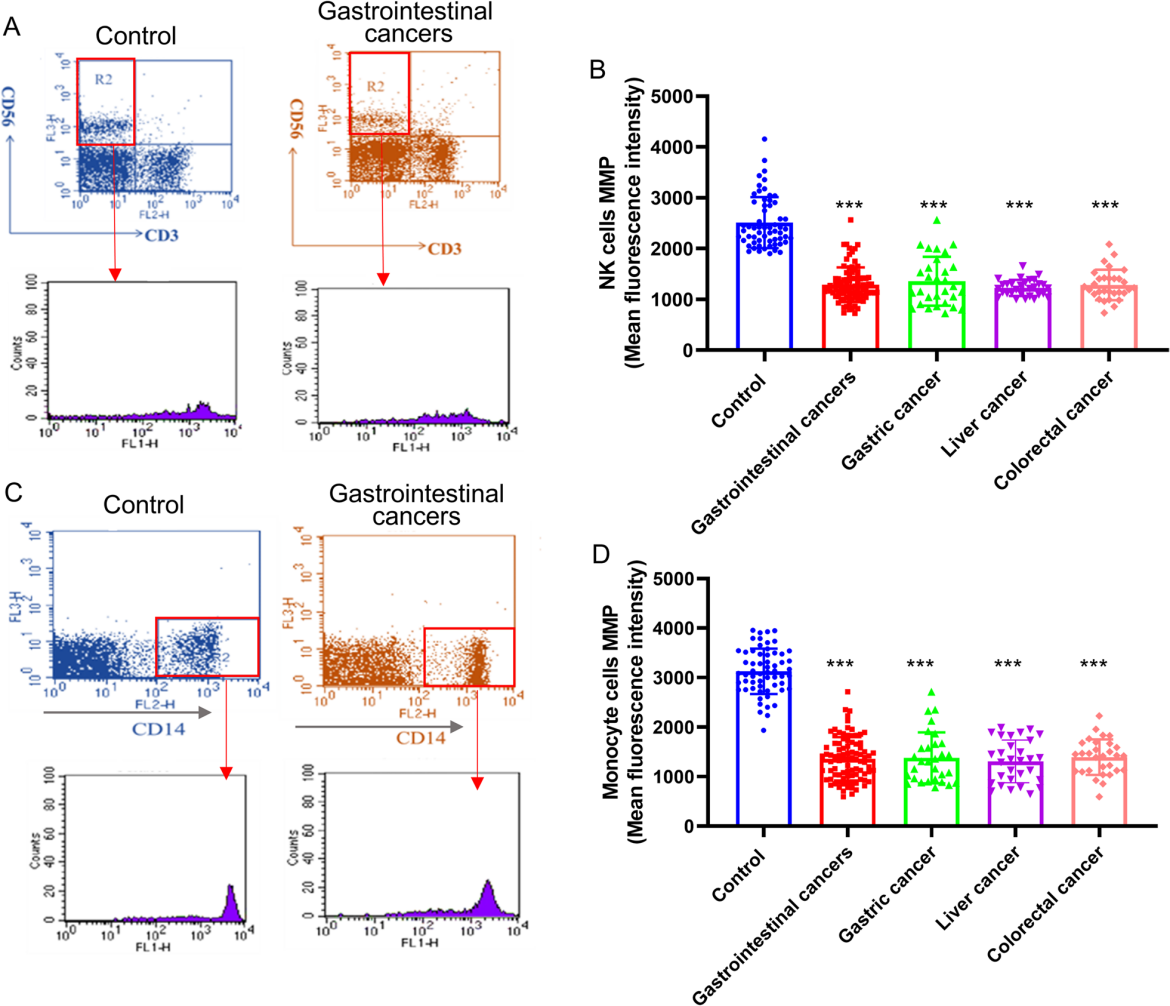
The mitochondrial membrane potential (MMP) of NK cells and monocytes in the primary gastrointestinal cancer patients without anti-tumor treatments and healthy people. **A** The MMP level of NK cells (CD3^−^CD56^+^) were detected by flow cytometric analysis. **B** The MMP level of T cells (CD3^−^CD56^+^) in the primary gastrointestinal cancer patients diagnosed for the first time and healthy people. **C** The MMP level of monocytes (CD14^+^) were detected by flow cytometric analysis. **D** The MMP level of monocytes (CD14^+^) in the primary gastrointestinal cancer patients diagnosed for the first time and healthy people. Values in the graphs represent the mean ± standard deviation; the symbol (***) indicates a significant increase compared with the control group (*P* < 0.001)

Mitochondrial membrane potential (ΔΨm) is a precise and robust indicator of the organelle status, proton pump and permeability transition, which plays a key role in vital mitochondrial functions. The ΔΨm was markedly reduced in PBMCs from the primary gastrointestinal cancer patients without anti-tumor treatments compared with that in the control group (Fig. 4A, B). We further analyzed the changes of MMP in different subgroup of immune cells. T cells and B cells in PBMCs were labelled with anti-CD3, anti-CD19 antibody and their ΔΨm was measured. We observed that the ΔΨm in CD3^+^ CD19^−^ T cells and CD3^−^ CD19^+^ B cells was significantly lower in the primary gastrointestinal cancer patients without anti-tumor treatments than that in the healthy donors (Fig. 4A, C and D). The ΔΨm of NK cells, which were labelled with CD3^−^CD56^+^, was markedly reduced in PBMCs from the primary gastrointestinal cancer patients without anti-tumor treatments (Fig. 5A, B). Also, the ΔΨm of CD14^+^ monocyte was significantly decreased in the gastrointestinal cancer patients without anti-tumor treatments (Fig. 5C, D). Moreover, the significant decrease of ΔΨm in immune cells from the primary gastrointestinal cancer patients without anti-tumor treatments was independent of the cancer subtypes, including gastric cancer, liver cancer and colorectal cancer (Figs. 4 and 5).

### Mitochondrial changes of PBMCs lead to the decrease and dysfunction of immune cells in the primary gastrointestinal cancer patients without anti-tumor treatments

**Fig. 6:**
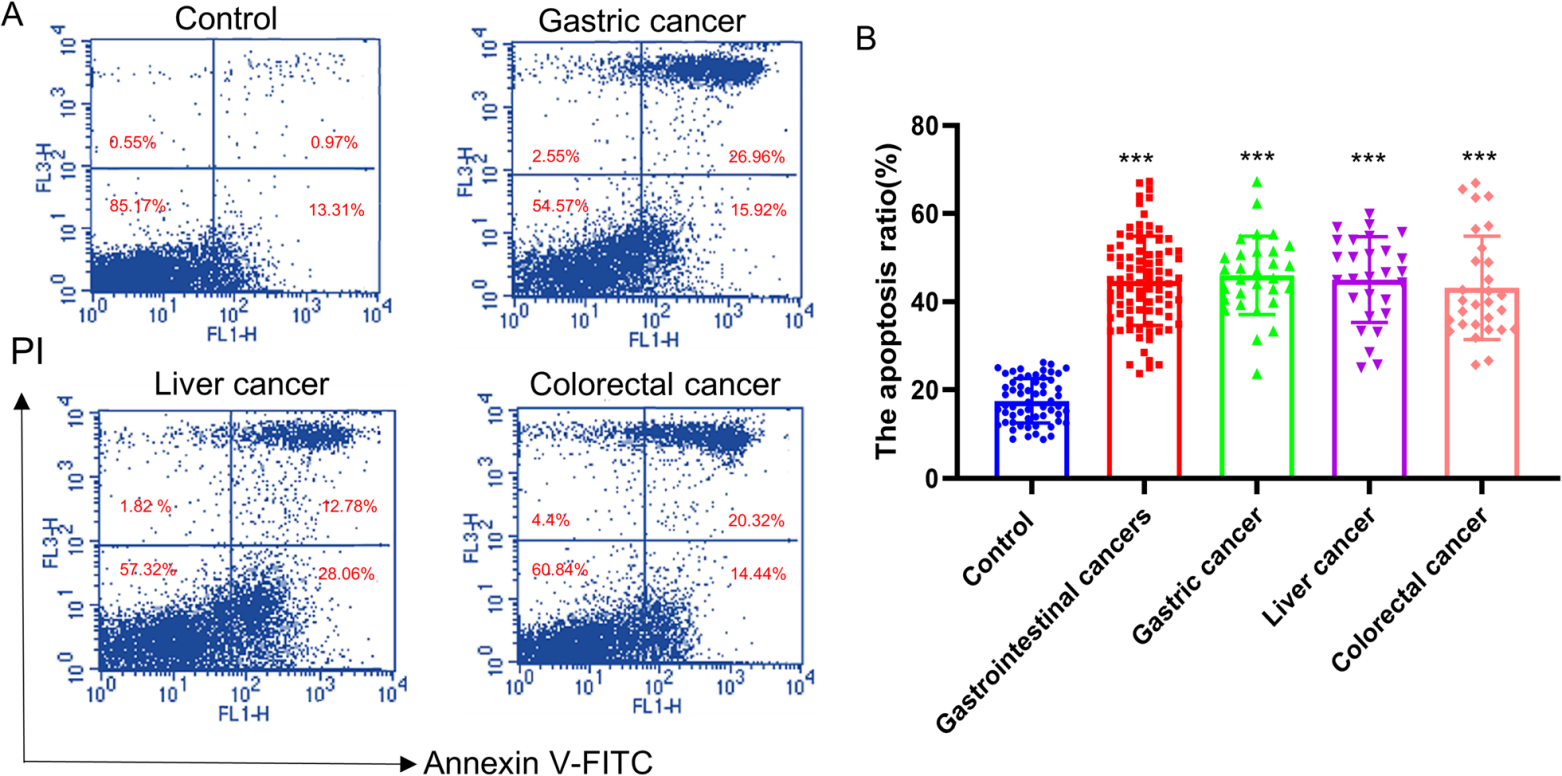
The apoptosis ratio of PBMCs in the primary gastrointestinal cancer patients without anti-tumor treatments and healthy people. **A** The apoptosis of PBMCs were detected by flow cytometric analysis. **B** The apoptosis ratio of PBMCs in the primary gastrointestinal cancer patients diagnosed for the first time and healthy people. Values in the graphs represent the mean ± standard deviation; the symbol (***) indicates a significant increase compared with the control group (*P* < 0.001)

**Fig. 7:**
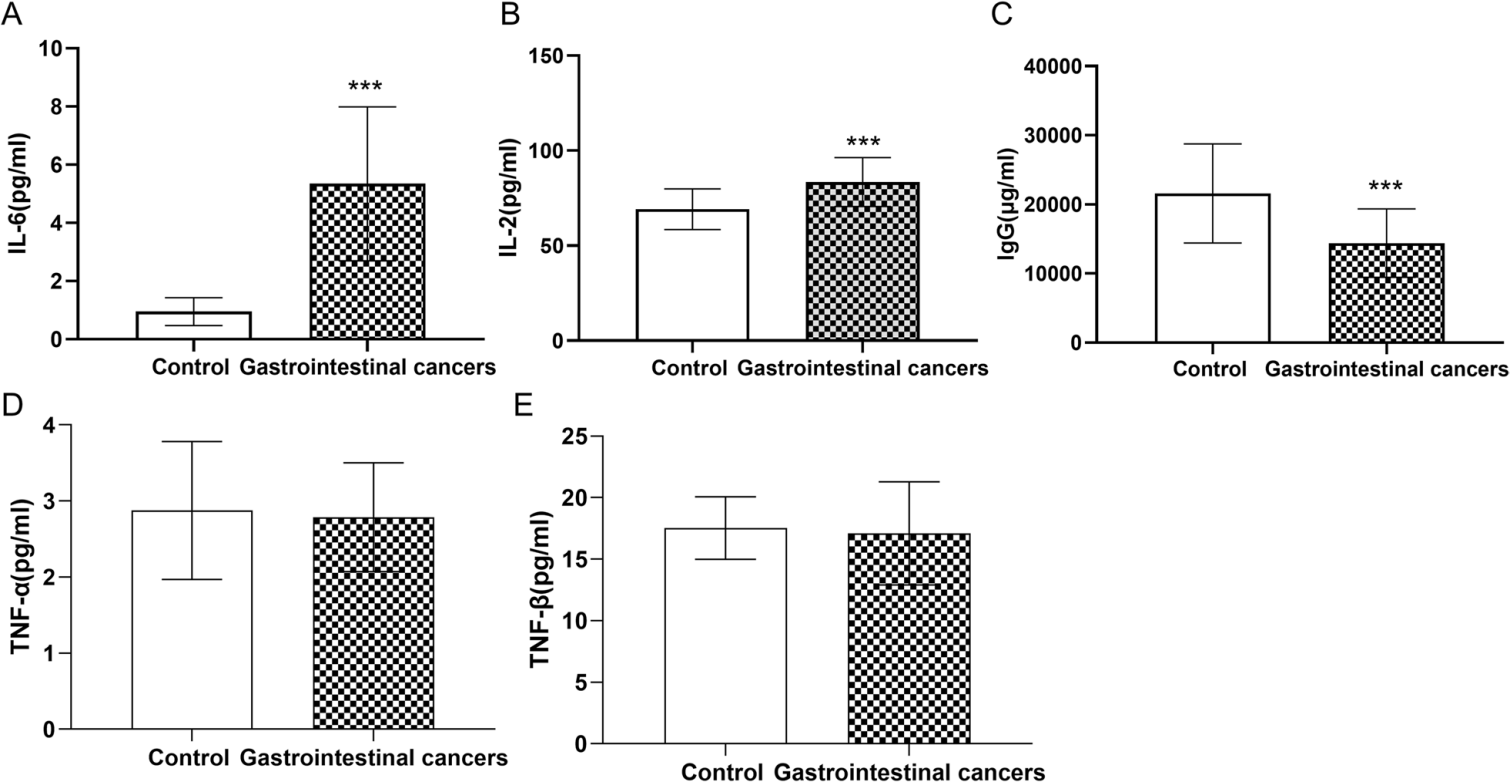
The level of Immune inflammatory factor in the primary gastrointestinal cancer patients without anti-tumor treatments and healthy people. **A** The level of IL-6 in plasma. **B** The level of IL-2 in plasma. **C** The level of IgG in plasma. **D** The level of TNF-α in plasma. **E** The level of TNF-β in plasma. Values in the graphs represent the mean ± standard deviation; the symbol (***) indicates a significant increase compared with the control group (*P* < 0.001)

In addition to the generation of ATP and ROS, mitochondria is also involved in the initiation of apoptosis. We examined the apoptosis ratio of PBMCs in the primary gastrointestinal cancer patients without anti-tumor treatments and healthy controls. Compared with the controls, the apoptosis ratio in PBMCs of patients with gastrointestinal cancers was significantly increased. And three cancer subtypes, such as gastric cancer, liver cancer and colorectal cancer, exhibited the same enhancement. (Fig. 6A, B). Immune cells secrete a variety of cytokines, which participate in the occurrence and development of cancer. Immune inflammatory factors and IgG were detected in the primary gastrointestinal cancer patients without anti-tumor treatments and healthy controls. Compared with the control group, the level of IL-2, IL-6 were significantly increased and IgG were significantly decreased in patients with primary gastrointestinal cancers (Fig. 7A–C). While, the levels of TNF-α and TNF-β was slightly changed between patients with primary gastrointestinal cancers and healthy donors, but there were no statistical significances (Fig. 7D, E), These results suggested that the apoptosis and secreted cytokines of immune cells were affected by the mitochondrial dysfunction in the primary gastrointestinal cancers without anti-tumor treatments.

## Discussion

Our study found that the total antioxidant capacity (T-AOC) and glutathione peroxidase (GSH-P_X_) activities of cancer patients decreased significantly, and the content of malondialdehyde (MDA) increased significantly, suggesting that there was an oxidation/antioxidation imbalance in the primary gastrointestinal cancer patients without anti-tumor treatments. These findings support that oxidative stress is related to the occurrence and development of various tumors, such as gastric cancer, colon cancer and bladder cancer [17–19]. Moreover, the level of ROS in PBMCs of gastrointestinal cancers was significantly increased. ROS, as an important metabolite of oxidative stress, can activate mitogen activated protein kinase (MAPK) signal pathway and promote tumor cell proliferation [20]. Also, the increase of ROS in tumor cells is positively associated with the downstream PI3K/Akt/mTOR signal transduction pathway [21]. Consistently, we found that up-regulated genes in tumor tissues of patients with gastrointestinal cancers were enriched in response to oxidative stress, PI3K-Akt and MAPK signaling pathway.

As the main place for energy generation, mitochondria are also the main site for the production of ROS [22, 23]. In this study, the level of mtROS in the PBMCs of the gastrointestinal cancer patients without anti-tumor treatments was also higher than that in the control group. Mitochondrial DNA itself lacks the protection of DNA binding protein or histone, so mitochondrial DNA is very vulnerable to oxygen free radical damage, resulting in mitochondrial biosynthesis disorder [24]. mtDNA encodes 13 protein components of the mitochondrial oxidative phosphorylation system, which are essential for aerobic ATP production. The mtND-1 copy number in PBMCs of the primary gastrointestinal cancer patients without anti-tumor treatments was lower than that in healthy donors, consistent with the decrease of ΔΨm and ATP. ΔΨm is the premise of maintaining mitochondrial oxidative phosphorylation and ATP production, and is necessary to maintain mitochondrial function [25]. The decrease of ΔΨm can not only inhibit cell energy metabolism, but also increase mitochondrial membrane permeability, release cytochrome oxidase C and cause apoptosis [26]. Based on the fact that ΔΨm is a precise and robust indicator of the mitochondrial status, we examined the similar changes in various subgroup of immune cells, such as T cell, B cell, NK cell and monocyte. The ATP, ΔΨm and mtDNA copy number of PBMCs was markedly reduced in PBMCs from the primary gastrointestinal cancer patients without anti-tumor treatments, indicating that mitochondrial oxidative stress of PBMCs, especially immune cells, caused mitochondrial dysfunction, which may be one of the main causes of primary gastrointestinal cancers.

Immune cells play important roles in tumor pathogenesis and progression. Both innate and adaptive immune cell types, effector molecules and pathways can collectively function as extrinsic tumor-suppressor mechanisms [27]. When the immune function is weakened, the ability of immune cells to clear tumor cells decreases. Meanwhile, tumor cells achieve immune escape by inducing active effector cell apoptosis and immune offset [28–30]. Based on data from our study, immune cells in primary gastrointestinal cancers take place the remarkable changes, in line with the inherited or acquired immunodeficiencies in cancer patients [27]. It has been confirmed that the number of CD4^+^ T cells increased in gastric cancer tissue and peripheral blood of patients with gastric cancer, while the number of CD8^+^ T cells decreased [31]. NK cells can prevent cancer development and avoid relapse following adoptive immunotherapy [32]. These results demonstrated that the composition and function of immune cells in patient’s peripheral blood are closely related to cancer occurrence and development.

Immune cells secrete a variety of cytokines (IL-2, IL-6, TNF-α, etc.) to participate in the regulation of tumorigenesis. B lymphocytes, known as antibody producers, mediate tumor cell destruction in the manner of antibody-dependent cell-mediated cytotoxicity [33]. The level of IgG in the primary gastrointestinal cancer patients without anti-tumor treatments significantly decreased, suggesting the inhibition of differentiation of B cells into IgG-producing cells during tumorigenesis. Additionally, IL-2, a T cell growth factor, plays a critical role in activating T cells, natural killer (NK) cells and macrophages in both the innate and adaptive immune system. Also, studies have shown that the persistence of activated CD8^+^ T cells is negatively impacted by the strength of interleukin 2 (IL-2) signaling[34]. IL-6 can not only promote tumor growth and inhibit tumor cell apoptosis, but also activate NF-κB. Here, the increase of IL-2, IL-6 were found in the primary gastrointestinal cancer patients without anti-tumor treatments. These results suggest that combination of mitochondrial dysfunction and cytokine expression changes of immune cells in the primary gastrointestinal cancers without anti-tumor treatments leads to the loss of the immune function.

The novelty of this study lies in that we for the first time discovered an association between mitochondrial function of immune cell in peripheral blood and primary gastrointestinal cancers without anti-tumor treatments. There are still some limitations in our study. We only focused on the changes of mitochondrial function of PBMCs in patients with primary gastrointestinal cancers before anti-tumor treatments. While, assessing therapy response or progression free survival or any other secondary endpoint to indicate a clinical relevance of immune cells mitochondrial function/dysfunction in gastrointestinal cancers is a very valuable study. The application of immune cells mitochondrial function/dysfunction into clinically relevant assessment will be further investigated in future.

## Conclusion

Collectively, the imbalance of oxidation and antioxidation was observed in the primary gastrointestinal cancer patients without anti-tumor treatments. The level of ROS and mtROS in the PBMCs of the primary gastrointestinal cancer patients without anti-tumor treatments was higher than that of control group. The decrease of ATP, mtDNA copy number and ΔΨm of PBMCs is associated with the primary gastrointestinal cancers without anti-tumor treatments. Also, the decreased levels of ΔΨm occurs in immune cells, including T cell, B cell, NK cell and monocyte. In addition, there were significant differences in the apoptosis ratio of PBMCs and the cytokine level between the primary gastrointestinal cancer patients without anti-tumor treatments and normal healthy donors. More importantly, these differences of mitochondrial function in immune cells were consistent among primary gastrointestinal cancers, such as liver cancer, gastric cancer and colorectal cancer. Further experimental studies are necessary to investigate the relationship between immune cells mitochondrial function/dysfunction and therapy response or progression free survival in gastrointestinal cancers.

## Data Availability

All data generated or analyzed during this study are included in this published article.

## References

[1] Sung H, Ferlay J, Siegel RL, Laversanne M, Soerjomataram I, Jemal A, Bray F (2021). Global cancer statistics 2020: GLOBOCAN estimates of incidence and mortality worldwide for 36 cancers in 185 countries. CA Cancer J Clin.

[2] Arina A, Corrales L, Bronte V (2016). Enhancing T cell therapy by overcoming the immunosuppressive tumor microenvironment. Semin Immunol.

[3] Bryant MK, Ward C, Gaber CE, Strassle PD, Ollila DW, Laks S (2020). Decreased survival and increased recurrence in Merkel cell carcinoma significantly linked with immunosuppression. J Surg Oncol.

[4] Kung PC, Goldstein G, Reinherz EL, Schlossman SF (1979). Pillars article: monoclonal antibodies defining distinctive human T cell surface antigens. Science.

[5] Liu F, Chun-Hua XU, Xie HY, Hospital NY (2014). Analysis of peripheral blood lymphocyte subsets in patients with small cell lung cancer. J Clin Pulm Med.

[6] Vivarelli M, Risaliti A (2011). Liver transplantation for hepatocellular carcinoma on cirrhosis: strategies to avoid tumor recurrence. World J Gastroenterol.

[7] Granito A, Muratori L, Lalanne C, Quarneti C, Ferri S, Guidi M, Lenzi M, Muratori P (2021). Hepatocellular carcinoma in viral and autoimmune liver diseases: role of CD4 + CD25 + Foxp3 + regulatory T cells in the immune microenvironment. World J Gastroenterol.

[8] Chen PL, Chen CF, Chen Y, Guo XE, Huang CK, Shew JY, Reddick RL, Wallace DC, Lee WH (2013). Mitochondrial genome instability resulting from SUV3 haploinsufficiency leads to tumorigenesis and shortened lifespan. Oncogene.

[9] Martínez-Reyes I, Cardona L, Kong H, Vasan K, McElroy G, Werner M, Kihshen H, Reczek C, Weinberg S, Gao P, Steinert E, Piseaux R, Budinger G, Chandel N (2020). Mitochondrial ubiquinol oxidation is necessary for tumour growth. Nature.

[10] Long Q, Yang K, Yang Q (2015). Regulation of mitochondrial ATP synthase in cardiac pathophysiology. Am J Cardiovasc Dis.

[11] Mcbride HM, Neuspiel M, Wasiak S (2006). Mitochondria: more than just a powerhouse. Curr Biol.

[12] Pereira CV, Lebiedzinska M, Wieckowski MR, Oliveira PJ (2012). Regulation and protection of mitochondrial physiology by sirtuins. Mitochondrion.

[13] Chamoto K, Chowdhury PS, Kumar A, Sonomura K, Matsuda F, Fagarasan S, Honjo T (2017). Mitochondrial activation chemicals synergize with surface receptor PD-1 blockade for T cell-dependent antitumor activity. Proc Natl Acad Sci USA.

[14] Schenk U, Frascoli M, Proietti M, Geffers R, Traggiai E, Buer J, Ricordi C, Westendorf AM, Grassi F (2011). ATP inhibits the generation and function of regulatory T cells through the activation of purinergic P2X receptors. Sci Signal.

[15] Research NCancerGAtlas, Weinstein JN, Collisson EA, Mills GB, Shaw KR, Ozenberger BA, Ellrott K, Shmulevich I, Sander C, Stuart JM (2013). The cancer genome atlas pan-cancer analysis project. Nat Genet.

[16] Tang Z, Kang B, Li C, Chen T, Zhang Z (2019). GEPIA2: an enhanced web server for large-scale expression profiling and interactive analysis. Nucleic Acids Res.

[17] Jelic MD, Mandic AD, Maricic SM, Srdjenovic BU (2021). Oxidative stress and its role in cancer. J Cancer Res Ther.

[18] Sajadimajd S, Khazaei M (2018). Oxidative stress and cancer: the role of Nrf2. Curr Cancer Drug Targets.

[19] Kundaktepe BP, Sozer V, Durmus S, Kocael PC, Kundaktepe FO, Papila C, Gelisgen R, Uzun H (2021). The evaluation of oxidative stress parameters in breast and colon cancer. Medicine.

[20] Hou XM, Zhang T, Da Z, Wu XA (2019). CHPF promotes lung adenocarcinoma proliferation and anti-apoptosis via the MAPK pathway. Pathol Res Pract.

[21] Fattahi S, Amjadi-Moheb F, Tabaripour R, Ashrafi GH, Akhavan-Niaki H (2020). PI3K/AKT/mTOR signaling in gastric cancer: epigenetics and beyond. Life Sci.

[22] Li ZY, Yang Y, Ming M, Liu B (2011). Mitochondrial ROS generation for regulation of autophagic pathways in cancer. Biochem Biophys Res Commun.

[23] Rigoulet M, Yoboue ED, Devin A (2011). Mitochondrial ROS generation and its regulation: mechanisms involved in H(2)O(2) signaling. Antioxid Redox Signal.

[24] Shu L, Hu C, Xu M, Yu J, He H, Lin J, Sha H, Lu B, Engelender S, Guan M, Song Z (2021). ATAD3B is a mitophagy receptor mediating clearance of oxidative stress-induced damaged mitochondrial DNA. EMBO J.

[25] Urbani A, Prosdocimi E, Carrer A, Checchetto V, Szabo I (2020). Mitochondrial ion channels of the inner membrane and their regulation in cell death signaling. Front Cell Dev Biol.

[26] Galluzzi L, Kepp O, Kroemer G (2016). Mitochondrial regulation of cell death: a phylogenetically conserved control. Microb Cell.

[27] Vesely MD, Kershaw MH, Schreiber RD, Smyth MJ (2011). Natural innate and adaptive immunity to cancer. Annu Rev Immunol.

[28] Koukourakis MI, Giatromanolaki A (2020). Tumor microenvironment, immune response and post-radiotherapy tumor clearance. Clin Transl Oncol.

[29] Renner K, Singer K, Koehl GE, Geissler EK, Peter K, Siska PJ, Kreutz M (2017). Metabolic hallmarks of tumor and immune cells in the tumor microenvironment. Front Immunol.

[30] Mortezaee K (2020). Immune escape: a critical hallmark in solid tumors. Life Sci.

[31] Kroemer M, Turco C, Spehner L, Viot J, Idirene I, Bouard A, Renaude E, Deschamps M, Godet Y, Adotevi O, Limat S, Heyd B, Jary M, Loyon R, Borg C (2020). Investigation of the prognostic value of CD4 T cell subsets expanded from tumor-infiltrating lymphocytes of colorectal cancer liver metastases. J Immunother Cancer.

[32] Malmberg KJ, Carlsten M, Bjorklund A, Sohlberg E, Bryceson YT, Ljunggren HG (2017). Natural killer cell-mediated immunosurveillance of human cancer. Semin Immunol.

[33] Liu J, Li Y, Lu Z, Gu J, Liang Y, Huang E, Wang Z, Zhang H, Wang L, Zhang D, Yu H, Liu R, Chu Y (2019). Deceleration of glycometabolism impedes IgG-producing B-cell-mediated tumor elimination by targeting SATB1. Immunology.

[34] Rajagopalan A, Berezhnoy A, Schrand B, Puplampu-Dove Y, Gilboa E (2017). Aptamer-targeted attenuation of IL-2 signaling in CD8(+) T cells enhances antitumor immunity. Mol Ther.

